# Effect of ERCC1 polymorphisms on the response to platinum-based chemotherapy: A systematic review and meta-analysis based on Asian population

**DOI:** 10.1371/journal.pone.0284825

**Published:** 2023-05-04

**Authors:** Xiaoqing Wu, Wenping Lu, Cuihong Jiang, Dongni Zhang, Weixuan Zhang, Yongjia Cui, Zhili Zhuo, Heting Mei, Ya’nan Wang, Mengfan Zhang, Shuntai Chen

**Affiliations:** 1 Guang’anmen Hospital, China Academy of Chinese Medical sciences, Beijing, China; 2 Guang’anmen Hospital South Campus, China Academy of Chinese Medical Sciences, Beijing, China; 3 Beijing University of Chinese Medicine, Beijing, China; Noakhali Science and Technology University Faculty of Pharmacy, BANGLADESH

## Abstract

**Background:**

Platinum-based chemotherapy is one of the most common treatments for many cancers; however, the effect of chemotherapy varies from individual to individual. Excision repair cross complementation group 1 (ERCC1) is widely recognized as a key gene regulating nucleotide excision repair (NER) and is closely associated with platinum response. Many studies have yielded conflicting results regarding whether ERCC1 polymorphisms can affect the response to platinum and overall survival (OS). Therefore, it is necessary to perform a meta-analysis of patients with specific races and cancer types.

**Methods:**

Eight databases (EMBASE, PubMed, Cochrane Library, Chinese National Knowledge Infrastructure, Scopus, VIP, China Biology Medicine disc and Wanfang databases) were searched. Results were expressed in terms of odds ratios (ORs), hazard ratios (HRs) and 95% CIs.

**Results:**

In this study, rs11615, rs2298881 and rs3212986 SNPs were studied. In the comparison between CT and TT on the response to platinum, esophageal cancer [I^2^ = 0%, OR = 6.18, 95% CI(1.89,20.23), P = 0.003] and ovarian cancer [I^2^ = 0%, OR = 4.94, 95% CI(2.21,11.04), P<0.001] showed that the rs11615 CT genotype predicted a better response. In the comparison between CC and TT, ovarian cancer [I^2^ = 48.0%, OR = 6.15, 95% CI (2.56,14.29), P<0.001] indicated that the CC genotype predicted a better response. In the meta-analysis of OS, the CC genotype was related to longer OS than TT in ovarian cancer [TT vs CC: I^2^ = 57.7%, HR = 1.71, 95% CI (1.18, 2.49), P<0.001].

**Conclusion:**

The ERCC1 rs11615 polymorphism was related to the response to platinum and OS, but the correlation is based on specific cancer types in the Asian population.

## Introduction

Platinum-based chemotherapy is the standard treatment for many cancers, such as ovarian cancer, gastric cancer, esophageal cancer, osteosarcoma, etc. Despite the clear clinical benefit of platinum, some patients still exhibit drug resistance and these patients are increasing with increasing treatment lines. Some studies have shown this phenomenon can be explained by the nucleotide excision repair (NER). DNA damage by platinum is the main anti-cancer mechanism of platinum [1, 2], and the damage can be repaired by the NER pathway [3]. Therefore, individual differences in NER capacity will lead to individual differences in response to platinum and thus to different prognoses.

It would certainly benefit patients and clinicians if we could predict the response of patients to platinum before they receive platinum-based chemotherapy. Platinum-sensitive patients receive platinum-containing chemotherapy, while platinum-insensitive patients receive non-platinum chemotherapy or other treatments. It can not only help clinicians to make decisions, but also reduce the pain caused by treatment. Excision repair cross complementation group 1 (ERCC1) component is a mammalian endonuclease that cleaves damaged DNA strands during the repair process of NER and interchain crosslinking. Detection of ERCC1 polymorphisms is expected to predict the efficacy of platinum-based chemotherapy. Two sub-pathways of NER, called global genome repair (GGR) and transcription-coupled repair (TCR), can be mentioned. DNA adducts including those induced by cisplatin are repaired by the nucleotide excision repair (NER) pathway [4]. ERCC1 is a subunit of the damaged 5′-side DNA endonuclease [5]. Following ERCC1-XPF nuclease mediated 5′ incision, the damaged oligonucleotide is excised and appears to bind to one or more components of the repair complex.

Many studies have found conflicting effects of ERCC1 polymorphisms on the platinum response. Gradually, it became clear that the cause of this conflict was ethnic differences. In studies examining the relationship between ERCC1 polymorphisms and the responsiveness of patients with non-small cell lung cancer treated with cisplatin, the results showed that ERCC1 polymorphisms were not associated with platinum responsiveness and there was no evidence of a potential prognostic role in a Caucasian population [6, 7]. While in Asian population, the T allele is a biomarker of low objective response, short progression free survival and overall survival (OS) in patients with gastric, osteosarcoma, ovarian and colorectal cancer [8–11]. So it is necessary to conduct a separate study on the impact of ERCC1 polymorphisms on platinum response and prognosis in Asian populations.

Currently, the most studied SNPs are rs11615, rs2298881 and rs3212986. Because the current study showed that the results of ERCC1 SNPs as a predictor of platinum treatment response and prognosis were contradictory, we performed a systemic review and meta-analysis to assess the evidence of effects of ERCC1 rs11615 C>T, rs2298881C>A and rs3212986 C>A SNPs on the efficacy of platinum-based chemotherapy and overall survival.

## Materials and methods

### 1. Literature search

The systematic review was performed according to the Meta-Analysis of Observational Studies in Epidemiology (i.e., MOOSE), Preferred Reporting Items for Systematic Reviews and Meta-analysis (PRISMA) statement and protocol. The systematic review protocol was registered with the international prospective register of systematic reviews (PROSPERO): CRD42022339301.

Two reviewers (CH J and ZL Z) independently searched databases including EMBASE, PubMed, Cochrane Library, Chinese National Knowledge Infrastructure (CNKI), Scopus, VIP, China Biology Medicine disc (CBM) and Wanfang from inception to June 2022. The search strategy is displayed in the S1 Table. The search time was from the establishment of the database to June 10, 2022. Two reviewers (CH J and DN Z) independently screened the titles and abstracts of all retrieved records to exclude irrelevant studies. The remaining studies were assessed by reading the full text. Any disagreement was resolved by consensus or by involving an arbiter (XQ W).

### 2. Study selection

Inclusion criteria: (1) Population: Asian patients who were included in detection (primary detection) or being followed for recurrent disease and had cancer recurrence detected in histopathology. Patients who were receiving chemotherapy including cisplatin, carboplatin, oxaliplatin or nedaplatin. (2) Intervention: ERCC1 polymorphisms, including rs11615 (CT vs CC vs TT), rs3212986 (AA vs CA vs CC) and rs2298881 (AA vs CA vs CC). (3) Control: each phenotypic data was served as a comparator with the other two (rs11615: CT vs CC vs TT; rs3212986: AA vs CA vs CC and rs2298881: AA vs CA vs CC). (4) Outcome: Primary outcome is served as the response to platinum-based chemotherapy in each SNP. After receiving platinum containing chemotherapy, patients with CR or PR were defined as having good response, while those with SD or PD were defined as having poor response. The secondary outcome was defined as OS for each SNP. OS was defined as the time from the observation point to death from any cause. (5) Study design: cohort and case control studies.

Exclusion criteria: All studies with mutated bases in gene fragments that did not meet the requirements listed in the inclusion criteria were excluded. We also excluded studies reporting data on laboratory developments tests as well as reviews, letters to editors, editorials, study protocols, case reports, brief correspondence, and articles published in languages other than English or Chinese. The references of all papers included were scanned for additional studies of interest.

### 3. Data extraction

Three investigators independently extracted the following information from the included articles: author name, publication year, country, chemotherapy regimen, number of patients, cancer species, number of good responses (CR+PR) and poor response (SD+PD), hazard ratio and 95% confidence interval between different genotypes and overall survival. The Newcastle‒Ottawa Quality Assessment Scale was used to assess the quality of each of the included studies. Any discrepancy was resolved by discussion or by involving an arbiter (XQ W).

### 4. Trial sequential analysis (TSA)

We assessed the risk of false positives or false negatives by TSA in a meta-analysis. Sequential monitoring boundaries were established to limit Type I error to 5% and Type II error to 20% to detect responsiveness to platinum and OS of patients under various models. When cumulative Z curves crossed conventional boundaries and trial sequential monitoring boundaries and the sample size reached the accrued information size (AIS), the expected intervention effect may reach a sufficient level of evidence. TSA was completed using the Stata, version 13.0, R package "foreign" and "ldBounds".

### 5. Statistical analysis

The effect estimates of interest were the OR (95%CI) in the meta-analysis of response to platinum and HR (95%CI) in the meta-analysis of overall survival. Studies exploring the response to platinum and overall survival of cancers were analyzed separately, and subgroup analyses were performed according to different cancer species. Assessment of heterogeneity was performed using Higgins I^2^; I^2^ greater than 50% and a P value less than 0.10 suggested significant heterogeneity, and a random effect model was applied; I^2^ less than 50% and a P value more than 0.10 suggested tolerable heterogeneity, and a fixed effect model was applied. Sensitivity analysis was performed by sequentially omitting each study to examine the robustness of the results. Potential publication bias was evaluated using a funnel plot (number of studies ≥ 10) and Begg’s and Egger’s tests. If significant publication bias existed, the trim and fill method was performed to validate the robustness of the meta-analysis results. Sensitivity analysis adopts the row-by-row division method. All statistical analyses were calculated via Stata, version 13.0 (StataCorp, College Station, TX). P values < 0.05 and Bonferroni-corrected p < 0.017 in studies with the response to platinum as the outcome (Results 2) and p values <0.05 and Bonferroni-corrected p < 0.025 in studies with OS as the outcome (Results 3), were defined as statistically significant, except those for heterogeneity.

## Results

### 1. Literature research

**Fig 1:**
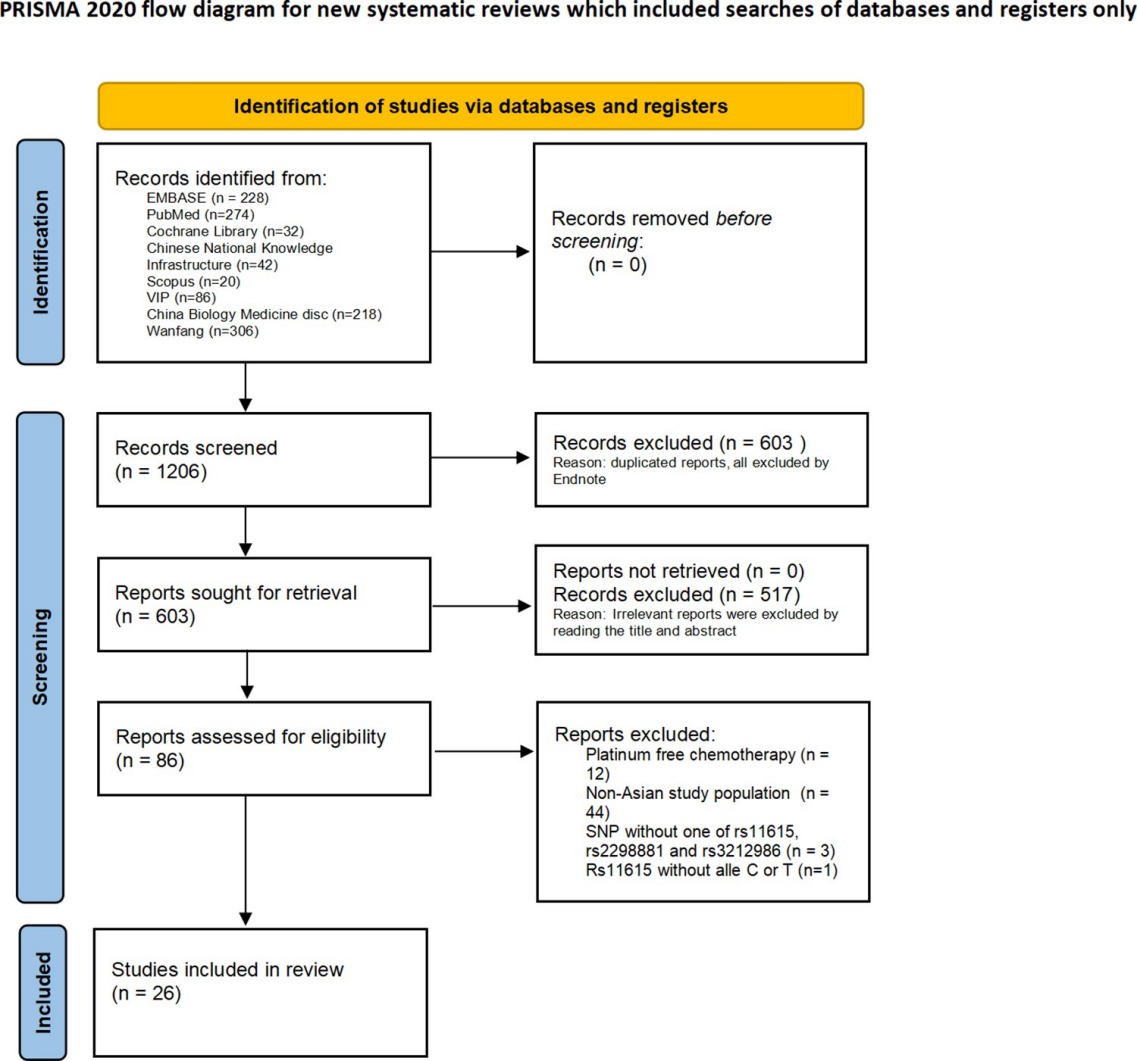
Plot of data collection procedure.

We identified 42 studies in CBM, 228 studies in Embase, 274 studies in the PubMed database, 32 studies in the Cochrane Library, 218 studies in the CNKI database, 86 studies in the VIP database, 20 studies in Scopus, and 306 studies in the Wanfang database. The flowchart of study selection is shown in Fig 1, and 27 studies were included in our meta-analysis. Among these studies, 26 studies reported the relationship between the ERCC1 rs11615 (C>T) polymorphism and platinum reactivity, 8 of which reported the effect of the rs11615 (C>T) polymorphism on OS. Six studies reported the relationship between the rs229981 (C>A) polymorphism and the response to platinum, 3 of which focused on the influence of the rs229981 (C>A) polymorphism on OS. Ten studies observed an association between the rs3212986 (C>A) polymorphism and the response to platinum, 6 of which also reported the rs3212986 (C>A) polymorphism and OS. The characteristics of these studies are summarized in Table 1. Newcastle‒Ottawa Scale (NOS) assessments are shown in Table 2.

**Table 1:** Characteristics of studies in the meta-analysis.

Study	Country	Cancer species	Drug	No.	Outcomes	Good response	Poor response	OS [HR(95%CI)]	SNPs
Huang X 2017 [12]	China	esophageal	docetaxel and cisplatin	50	TR,PFS,OS	CC:17 CT:10 TT:3	CC:7 CT:6 TT:7	CC: Ref. CT: 1.27 (0.39–4.12) TT: 12.96 (3.08‑54.61)	rs11615
Qi BL 2013 [13]	China	ovarian	Platinum-based chemotherapy	210	TR,PFS,OS	CC:78 CT:67 TT:2	CC:38 CT:26 TT:9	CC: Ref. TT: 2.22 (1.06‑4.06)	rs11615
Yang SY 2017 [15]	China	ovarian	Cisplatin or carboplatin combined with cyclophosphamide or paclitaxel	209	TR,PFS(rs11615),OS(rs11615)	rs11615: CC:70 CT:31 TT:1 rs3213986: CC:43 CA:22 AA:6	rs11615: CC:28 CT:67 TT:12 rs3213986: CC:74 CA:55 AA:9	rs11615: CC: Ref. TT: HR = 2.87,95%CI(1.38–5.96)	rs11615, rs3213986
Huo XY 2010 [26]	China	ovarian	Taxol/docetaxel+cisplatin/carboplatin/oxaliplatin	370	TR,PFS,OS	CC:104 CT:82 TT:6	CC:43 CT:29 TT:16	CC: Ref. CT: 2.13(1.02–4.58) TT: 1.12(0.59–1.75)	rs11615
Bao YX 2020 [16]	China	ovarian	taxol plus cisplatin	559	TR	rs11615: CC:115 CT:55 TT:9 rs3213986: CC:100 CA:64 AA:15 rs2298881: CC:53 CA:96 AA:30	rs11615: CC:209 CT:124 TT:47 rs3213986: CC:180 CA:132 AA:68 rs2298881: CC:120 CA:195 AA:65	-	rs11615, rs3212986, rs2298881
Cao ZH 2015 [17]	China	osteosarcoma	cisplatin-based treatment	186	TR,OS	rs11615: CC:23 CT:46 TT:43 rs3213986: CC:54 CA:45 AA:61 rs2298881: CC:22 AC:29 AA:61	rs11615: CC:8 CT:28 TT:38 rs3213986: CC:40 CA:29 AA:50 rs2298881: CC:6 CA:18 AA:50	rs3212986: CC: Ref. CA: 0.85 (0.41–1.76) AA: 0.60 (0.13–2.14)	rs11615, rs3212986, rs2298881
Ji WP 2015 [18]	China	osteosarcoma	Cisplatin-based chemotherapy	214	TR	CC:27 CT:54 TT:52	CC:9 CT:31 TT:41	-	rs11615
Sun YJ 2015 [19]	China	osteosarcoma	Cisplatin-based chemotherapy	172	TR,OS	rs11615: CC:81 CT:16 TT:10 rs2298881: AA:50 AC:48 CC:9	rs11615: CC:35 CT:15 TT:15 rs2298881: AA:16 AC:34 CC:15	rs11615: CC: Ref. CT: 1.49 (0.50–4.03) TT: 3.36(1.19–9.16) rs2298881: AA: Ref. AC: 1.61 (0.66–4.07) CC: 3.57 (1.10–11.35)	rs11615, rs2298881
Li JK 2014 [20]	China	gastric	5-Fu/capecitabine+ cisplatin/ oxaliplatin/ paclitaxel/ docetaxel	236	TR,OS	rs11615: CC:91 CT:76 TT:28 rs3213986: CC:85 CA:71 AA:39 rs2298881: CC:16 AC:48 AA:131	rs11615: CC:47 CT:54 TT:30 rs3213986: CC:51 CA:50 AA:30 rs2298881: CC:13 AC:35 AA:83	rs3212986: CC: Ref. CA: 1.28 (0.74–2.22) AA: 1.15 (0.59–2.22) rs2298881: AA: Ref. AC: 1.20 (0.68–2.10) CC: 1.06 (0.42–2.55)	rs11615, rs3212986, rs2298881
Yang LM 2012 [21]	China	osteosarcoma		187	TR	CC:47 CT:38 TT:12	CC:46 CT:35 TT:9	CC: Ref. CT: 0.81(0.40–1.33) TT: 0.74 (0.28–1.94)	rs11615
Hong W 2013 [22]	China	lung	gemcitabine/platinum	135	TR	rs11615: CC:20 CT:17 TT:2 rs3212986: CC:17 CA:19 AA:3	rs11615: CC:42 CT:48 TT:6 rs3212986: CC:43 CA:39 AA:14	-	rs11615, rs3212986
Cheng J 2012 [23]	China	lung	cisplatin+ vinorelbine or DDP + paclitaxel	142	TR	CC:35 CT:10 TT:2	CC:51 CT:37 TT:7	-	rs11615
Park SR 2011 [24]	Korea	gastric	Cisplatin+S-1	108	TR	rs11615: CC:35 CT:20 TT:3 rs3212986: AA:7 CA:21 CC:29	rs11615: CC:29 CT:17 TT:4 rs3212986: AA:1 CA:17 CC:33	rs11615: CC:Ref. CT:0.940(0.556–1.587) TT: 1.918 (0.748–4.919) rs3212986: AA: Ref. AC: 1.750 (0.578–5.304) CC: 1.988 (0.697–5.670)	rs11615, rs3212986
Li F 2010 [25]	China	lung	Vinorelbine plus DDP/CBP or Gemcitabine plus DDP/CBP or Taxol/Docetaxel plus DDP/ CBP	115	TR	rs11615: CC:13 CT:15 TT:2 rs3212986: CC:21 CA:6 AA:3	rs11615: CC:60 CT:25 TT:0 rs3212986: CC:30 CA:42 AA:13	-	rs11615, rs3212986
Chen SJ 2010 [26]	China	lung	cisplatinbased chemotherapy (NP、GP、TP)	95	TR	CC:25 CT:18 TT:2	CC:25CT:23 TT:2	-	rs11615
Liang J 2010 [27]	China	colon	Oxaliplatin+calcium folinate+5-fluorouracil/oxaliplatin+xeloda	113	TR	CC:44CT:29 TT:8	CC:11CT:14 TT:7	-	rs11615
Ma BB 2009 [28]	China-Hong Kong	nasopharyngeal	gemcitabine+oxaliplatin	29	TR	CC:11 CT:5 TT:1	CC:5 CT:4 TT:1	-	rs11615
Chung CH 2014 [59]	Korea	cervical	5-fluorouracil (5-FU)/platinum agent with or without adriamycin and etoposide /cisplatin.	35	TR	CC:16 CT:9 TT:3	CC:4 CT:3 TT:0	-	rs11615
Ryu JS 2004 [30]	Korea	Lung	cisplatin+paclitaxel/cisplatin+gemcitabine/cisplatin+docetaxel	109	TR	CC:29 CT:18 TT:4	CC:25 CT:27 TT:4	-	rs11615
Su D 2007 [31]	China	lung	vinorelbine+cisplatin /gemcitabine +cisplatin/taxol+cisplatin/radiotherapy	230	TR	CC:30CT:21 TT:3	CC:47CT:21 TT:3	-	rs11615
Zhang Q 2015 [32]	China	osteosarcoma	Cisplatin	260	TR	rs11615: CC:31 CT:68 TT:53	rs11615: CC:7 CT:41 TT:60	-	rs11615
Yu X 2015 [33]	China	esophageal	taxanes plus platinum/fluoropyrimidine plus platinum/paclitaxel plus platinum/irinotecan plus platinum	118	TR	CC:19 CT:28 TT:2	CC:33 CT:13 TT:9	-	rs11615
Zhong G 2015 [34]	China	gastric	Folfox	263	TR,OS	rs11615: CC:74 CT:57 TT:21 rs3212986: CC:74 CA:53 AA:25	rs11615: CC:38 CT:47 TT:26 rs3212986: CC:61 CA:36 AA:14	rs11615:CC: Ref.CT:1.56 (0.80–3.03)TT:2.72 (1.22–6.01)rs3212986:CC: Ref.CA:1.13 (0.60–2.14) AA:1.38 (0.58–3.13)	rs11615 rs3212986
Chen ZH 2014 [38]	China	gastric	5-Fu/capecitabine+ cisplatin/ oxaliplatin/ paclitaxel/ docetaxel	255	TR,OS	AA:98 AC:34 CC:24	AA:67 AC:24 CC:8	AA:Ref. AC: 0.85 (0.44–1.64) CC: 0.83 (0.35–1.93)	rs2298881
Zheng DL 2016 [35]	China	gastric	platinum-based chemotherapy	246	TR,OS	rs11615: CC:12 CT:58 TT:72 rs3212986: CC:80 CA:56 AA:6	rs11615: CC:10 CT:36 TT:36 rs3212986: CC:33 CA:37 AA:13	rs11615: TT: Ref. TC: 1.10 (0.56–2.13) CC: 1.27 (0.39–3.73) rs3212986: CC: Ref. CA: 1.60 (0.81–3.16) AA: 5.10 (1.63–16.17)	rs11615, rs3212986
Mo J 2015 [36]	China	gastric	platinum-based chemotherapy	228	TR,OS	rs11615: CC:14 CT:64 TT:75 rs3212986: CC:88 CA:60 AA:5	rs11615: CC:8 CT:36 TT:31 rs3212986: CC:30 CA:39 AA:6	rs11615: TT: Ref. TC: 1.03 (0.53–2.00) CC: 1.05 (0.30–3.17) rs3212986: CC: Ref. CA: 1.40 (0.72–2.71) AA: 6.19 (1.42–30.60	rs11615, rs3212986
Bai Y 2015 [37]	China	gastric	platinum-based chemotherapy	270	TR,OS	rs11615: CC:12 TC:58 TT:72 rs2298881: CC:75 AC:83 AA:18	rs11615: CC:10 TC:36 TT:36 rs2298881: CC:31 AC:47 AA:15	rs11615: TT: Ref. TC: 1.12 (0.65–1.93) CC: 1.22 (0.51–2.05) rs2298881: CC: Ref. AC: 1.10 (0.63–1.91) AA: 1.33 (0.56–3.13)	rs11615, rs2298881

**Table 2:** Quality assessment of the included studies.

Study ID	Selection				Comparability	Outcome			Total scores
	Representativeness of the exposed cohorts	Selection of the non-exposed cohort	Ascertainment of exposure	Demonstration that outcome of interest was not present at start of study	Cohorts on the basis of the design or analysis	Assessment of outcome	Was follow-up long enough for outcomes to occur	Adequacy of follow-up of cohorts	
Huang X 2017 [12]	☆	☆	☆	☆	☆☆	☆	☆	☆	9
Qi BL 2013 [13]	☆	☆	☆	☆	☆☆	☆	☆	☆	9
Yang SY 2017 [15]	☆	☆	☆	☆	☆☆	☆	☆	-	8
Huo XY 2010 [26]	☆	☆	☆	☆	-	☆	-	-	5
Bao YX 2020 [16]	-	☆	☆	☆	☆☆	☆	-	-	6
Cao ZH 2015 [17]	☆	☆	☆	☆	☆	☆	☆	-	7
Ji WP 2015 [18]	☆	☆	☆	☆	☆	☆	☆	-	7
Sun YJ 2015 [19]	☆	☆	☆	☆	☆☆	☆	☆	-	8
Li JK 2014 [20]	-	☆	☆	☆	☆☆	☆	☆	-	7
Yang LM 2012 [21]	☆	☆	☆	☆	☆	☆	-	-	6
Hong W 2013 [22]	-	☆	☆	☆	☆	☆	☆	-	6
Cheng J 2012 [23]	☆	☆	☆	☆	☆☆	☆	-	-	7
Park SR 2011 [24]	☆	☆	☆	☆	☆	☆	-	-	6
Li F 2010 [25]	☆	☆	☆	☆	☆☆	☆	-	-	7
Chen SJ 2010 [26]	☆	☆	☆	☆	☆☆	-	☆	-	7
Liang J 2010 [27]	☆	☆	☆	☆	☆☆	☆	☆	-	8
Ma BB 2009 [28]	☆	☆	☆	☆	☆☆	☆	☆	-	8
Chung CH 2014 [59]	☆	☆	☆	☆	-	☆	-	☆	6
Ryu JS 2004 [30]	☆	☆	☆	☆	☆☆	☆	-	-	7
Su D 2007 [31]	☆	☆	☆	☆	☆☆	☆	☆	-	8
Zhang Q 2015 [32]	☆	☆	☆	☆	☆☆	☆	☆	☆	9
Yu X 2015 [33]	-	☆	☆	☆	☆	☆	-	-	5
Zhong G 2015 [34]	☆	☆	☆	☆	☆	☆	☆	-	7
Chen ZH 2014 [38]	☆	☆	☆	☆	☆	☆	-	-	6
Zheng DL 2016 [35]	☆	☆	☆	☆	☆	☆	-	-	6
Mo J 2015 [36]	☆	☆	☆	☆	☆	☆	☆	-	7
Bai Y 2015 [37]	☆	☆	☆	☆	☆☆	☆	☆	-	8

### 2. Response to platinum‑based chemotherapy

#### 2.1 The rs11615 polymorphism

**Fig 2:**
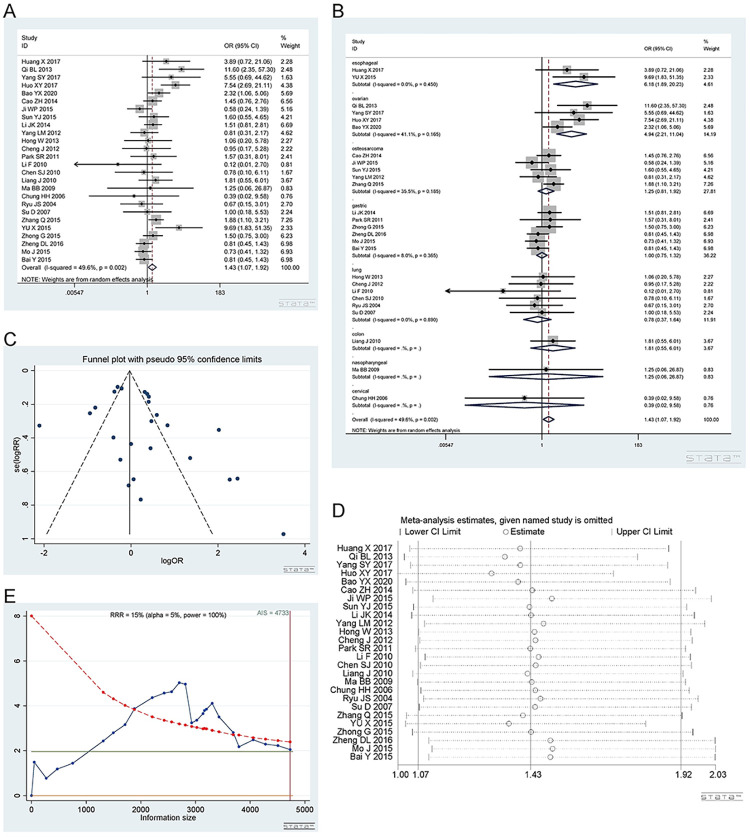
Meta-analysis of the response to platinum-based chemotherapy in the rs11615 dominant model. (a) Results of the response to platinum-based chemotherapy in all cancer types. (b) Results of the sensitivity analysis. (c) Results of subgroup analysis of the response to platinum-based chemotherapy according to cancer type. (d) The funnel plot of publication bias. (e) TSA on the response to platinum. The x-axis ticks refer to the studies added sequentially into the analysis. The blue curve was the adjusted Z-curve, and it crossed the dark-green horizontal line (conventional boundary for α = 0.05). The vertical red line shows the required information size—the needed sample size for a robust result with sufficient statistical power. The analysis included 4889 participants, which exceeded the required information size (n = 4733).

*2*.*1*.*1 Dominant model (CT+TT vs CC)*. The rs11615 polymorphism has a wild-type C allele and a variant T allele. A total of 26 studies [12–37], involving 4889 patients were included. There were 4 studies on ovarian cancer, 5 on osteosarcoma, 2 on esophageal cancer, 6 on gastric cancer, 6 on lung cancer, 1 on nasopharyngeal, 1 on cervical cancer and 1 on colon cancer. CT+TT vs CC: I^2^ = 50.4%, random effect model was used, OR = 0.81, 95% CI (0.66,0.99), P = 0.041, as shown in Fig 2(A). Sensitivity analysis showed unreliable results (Fig 2(B)). The results of Meta-regression showed that different cancer types may be the source of heterogeneity, especially colon cancer and osteosarcoma, with p-values of 0.088 and 0.089, respectively. Subsequently, subgroup analysis was performed according to cancer type, which showed that the ERCC1 rs11615 CT+TT genotype in ovarian cancer was related to a better platinum response. CT+TT vs CC: I^2^ = 0%, subtotal OR = 0.77, 95%CI(0.60, 0.97), P = 0.030, while esophageal, lung, gastric, nasopharyngeal, cervical cancer and osteosarcoma were not related. The results of the subgroup analysis are shown in Fig 2(C). It should be noted that there was only one study in the colon, nasopharyngeal and cervical cancer groups.

The funnel plot was basically symmetrical (Fig 2(D)), and Begg’s test and Egger’s test showed P = 0.243 and 0.251, respectively, indicating no publication bias. The TSA results showed that the enrolled studies were adequate (Fig 2(E)).

**Fig 3:**
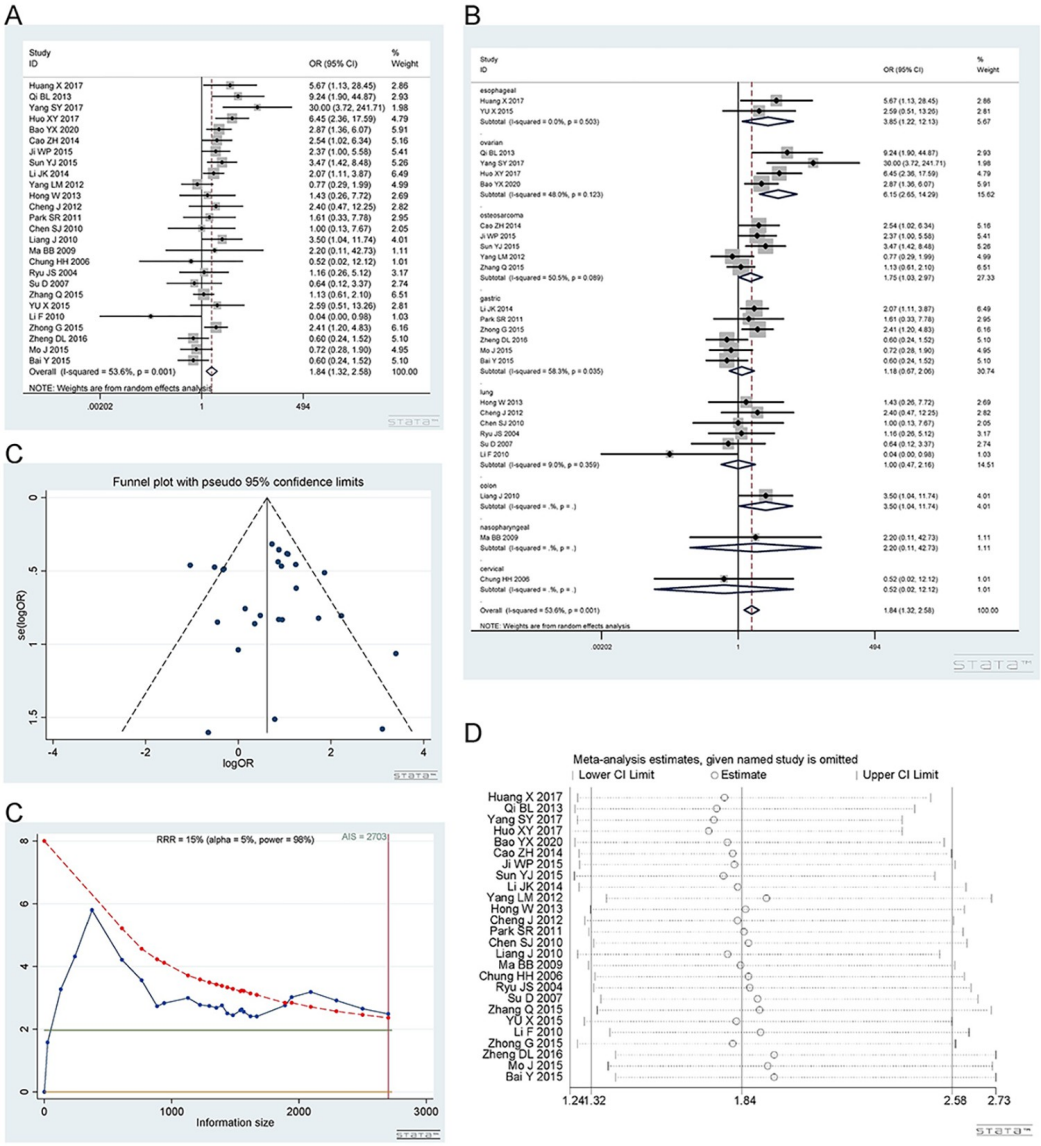
Meta-analysis of the response to platinum-based chemotherapy in the rs11615 heterozygous model. (a) Result of the response to platinum-based chemotherapy in all cancer types. (b) Result of subgroup analysis of the response to platinum-based chemotherapy according to cancer types. (c) The funnel plot of publishing bias. (d) Result of the sensitivity analysis. (e) TSA on the response to platinum. The analysis included 4889 participants, which exceeded the required information size (n = 2703).

*2*.*1*.*2 Heterozygous model (CT vs TT)*. Number of studies and people involved in the study as described in 2.1.1. The ERCC1 rs11615 polymorphism CT genotype was related to the response to platinum-based chemotherapy. Heterozygous model (CT vs TT): I^2^ = 52.0%, random effect model was used, OR = 1.43, 95% CI(1.07,1.92), P = 0.016, as shown in Fig 3(A). In the subgroup analysis, esophageal cancer [I^2^ = 0%, OR = 6.18, 95% CI (1.89, 20.23), P = 0.003] and ovarian cancer [I^2^ = 0%, OR = 4.94, 95% CI (2.21, 11.04), P<0.001] showed that the rs11615 CT genotype predicted a better response to platinum. In gastric cancer [I^2^ = 8.0%, OR = 1.00, 95%CI(0.75,1.32), P = 0.975], lung cancer [I^2^ = 0%, OR = 0.78, 95%CI(0.37,1.64), P = 0.509], colon cancer [OR = 1.81, 95%CI(0.55,6.01), P = 0.331], nasopharyngeal [OR = 1.25,95%CI(0.06,26.87)], cervical cancer [OR = 0.39,95%CI(0.02,9.58)] and osteosarcoma [I^2^ = 35.5%, OR = 1.25, 95%CI(0.81,1.92), P = 0.331], CT genotype seems to be not related to the response to platinum. The forest plot is shown in Fig 3(B). It should be noted that there was only one study in the colon, nasopharyngeal and cervical cancer groups.

The funnel plot was basically symmetrical (Fig 3(C)). Begg’s test and Egger’s test showed that P = 0.675 and 0.110, respectively, indicating that there was no publication bias. The sensitivity analysis showed that the result was reliable (Fig 3(D)). The TSA results showed that the enrolled studies were adequate (Fig 3(E)).

**Fig 4:**
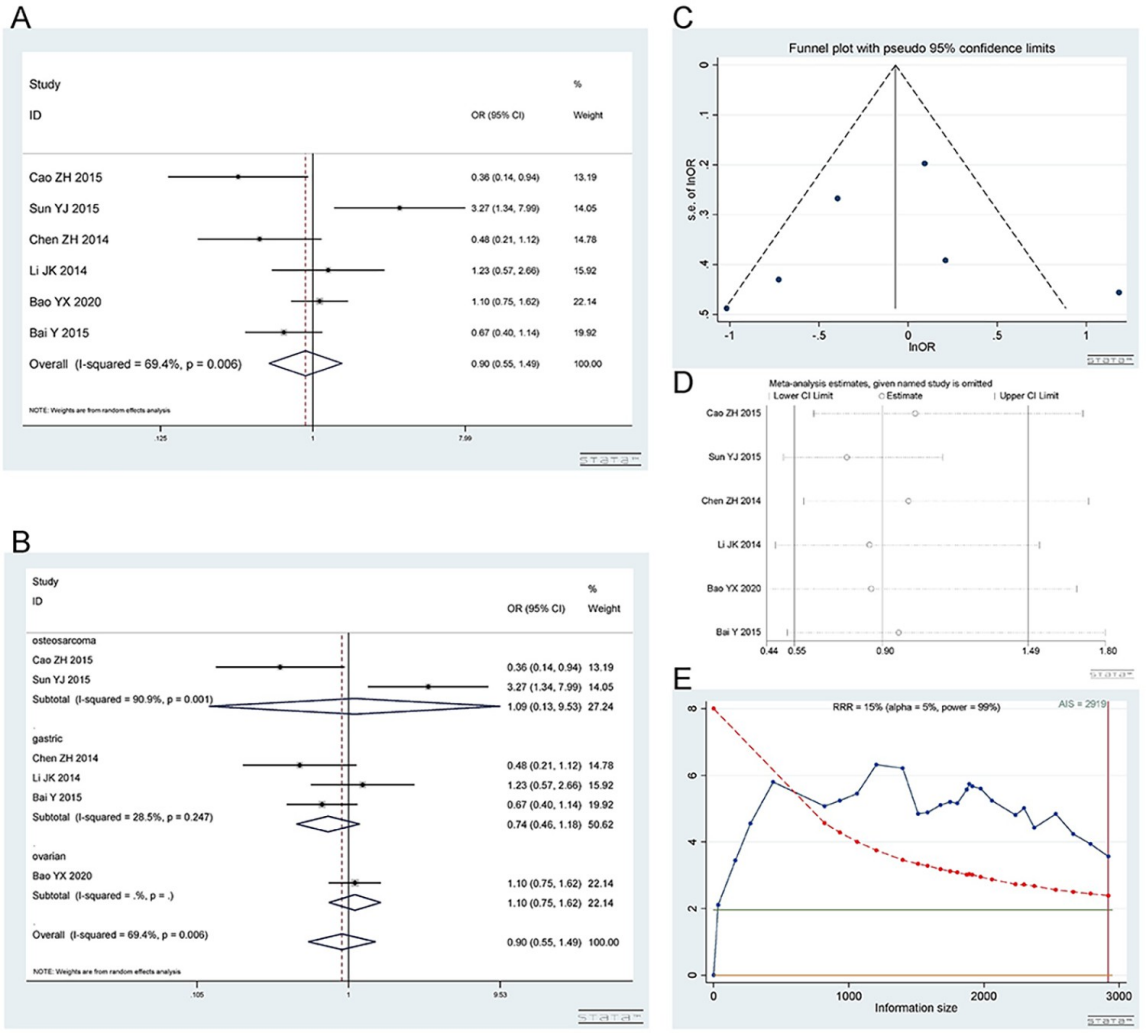
Meta-analysis of the response to platinum-based chemotherapy in the rs11615 homozygous model. (a) Result of the response to platinum-based chemotherapy in all cancer species. (b) Result of subgroup analysis of the response to platinum-based chemotherapy according to cancer species. (c) The funnel plot of publishing bias. (d) Result of the sensitivity analysis. (e) TSA on the response to platinum. The analysis included 4889 participants, which exceeded the required information size (n = 2919).

*2*.*1*.*3 Homozygous model (CC vs TT)*. Number of studies and people involved in the study as described in 2.1.1. ERCC1 rs11615 CC genotype was related to a good response to platinum-based chemotherapy. Homozygous model (CC vs TT): I^2^ = 53.6%, random effect model was used, OR = 1.84, 95%CI(1.32,2.58), P<0.001, as shown in Fig 4(A). In the subgroup analysis, esophageal cancer [I^2^ = 0%, OR = 3.85, 95%CI (1.22,12.13), P = 0.021], ovarian cancer [I^2^ = 48.0%, OR = 6.15, 95% CI (2.56,14.29), P<0.001] and colon cancer [OR = 3.50, 95% CI (1.04,11.74), P = 0.043] showed that CC predicted a better response to platinum than the TT genotype. In gastric cancer [I^2^ = 58.3%, OR = 1.18, 95%CI(0.67,2.06), P = 0.568], lung cancer [I^2^ = 0%, OR = 1.00, 95%CI(0.47,2.16), P = 0.993], nasopharyngeal [OR = 2.20,95%CI(0.11,42.73)], cervical cancer [OR = 0.52,95%CI(0.02,12.12)] and osteosarcoma [I^2^ = 50.5%, OR = 1.75, 95%CI(1.03,2.97), P = 0.039], CC vs TT seems to be not related to the response to platinum. The subgroup plot is shown in Fig 4(B). It should be noted that there was only one study in the colon, nasopharyngeal and cervical cancer groups.

The funnel plot was basically symmetrical (Fig 4(C)). Begg’s test and Egger’s test showed that P = 0.675 and 0.175, respectively, indicating that there was no publication bias. The sensitivity analysis showed that the result was reliable (Fig 4(D)). The TSA results showed that the enrolled studies were adequate (Fig 4(E)).

#### 2.2 The rs2298881 A/C polymorphisms

**Fig 5:**
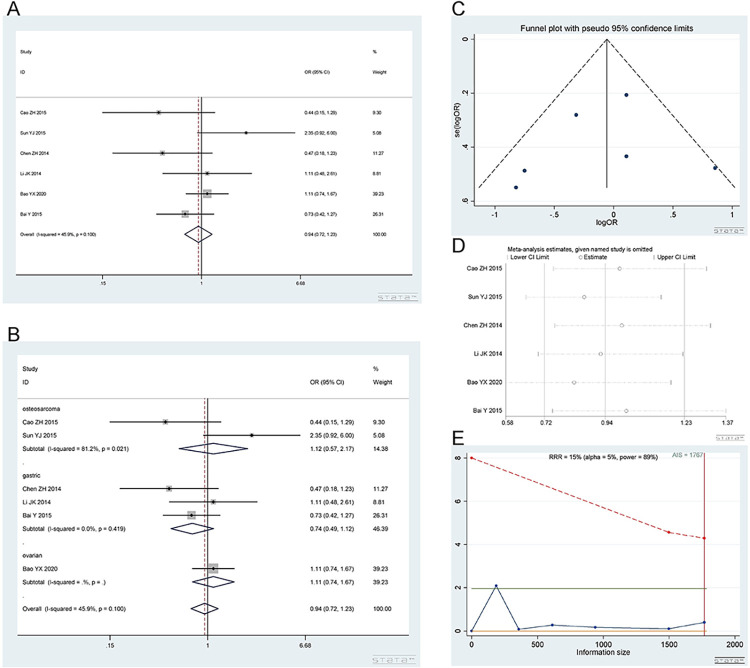
Meta-analysis of the response to platinum-based chemotherapy in the rs2298881 dominant model. (a) Result of the response to platinum-based chemotherapy in all cancer types. (b) Result of subgroup analysis of the response to platinum-based chemotherapy according to cancer type. (c) The funnel plot of publishing bias. (d) Result of the sensitivity analysis. (e) TSA on the response to platinum. The analysis included 1678 participants, which does not exceed the required information size (n = 1767).

*2*.*2*.*1 Dominant model (CA+AA vs*. *CC)*. The rs2298881 polymorphism has a wild-type C allele and variant A allele. A total of 6 studies [16, 17, 19, 20, 37, 38], involving 1678 patients were included. There were 3 studies on gastric cancer, 2 on osteosarcoma and 1 on ovarian cancer. The CA+AA and CC genotypes showed no difference in platinum sensitivity. Dominant model (CA+AA vs CC): I^2^ = 69.4%, random effect model was used, OR = 0.90, 95% CI (0.55,1.49), P = 0.693, as shown in Fig 5(A). In the subgroup analysis, the CA+AA vs CC genotype was not related to platinum response in the gastric cancer [I^2^ = 28.5%, OR = 0.74, 95% CI (0.46,1.18), P = 0.204], osteosarcoma [I^2^ = 90.9%, OR = 1.09, 95% CI (0.13,9.53), P = 0.936] and ovarian cancer [OR = 1.10, 95% CI (0.75,1.62), P = 0.638] groups, as shown in Fig 5(B).

The funnel plot was basically symmetrical (Fig 5(C)). Begg’s test and Egger’s test showed that P = 0.452 and 0.829, respectively, indicating that there was no publication bias. The sensitivity analysis showed that the result was robust (Fig 5(D)). The TSA results showed that the enrolled studies were not adequate and need to be verified by more relevant studies (Fig 5(E)).

**Fig 6:**
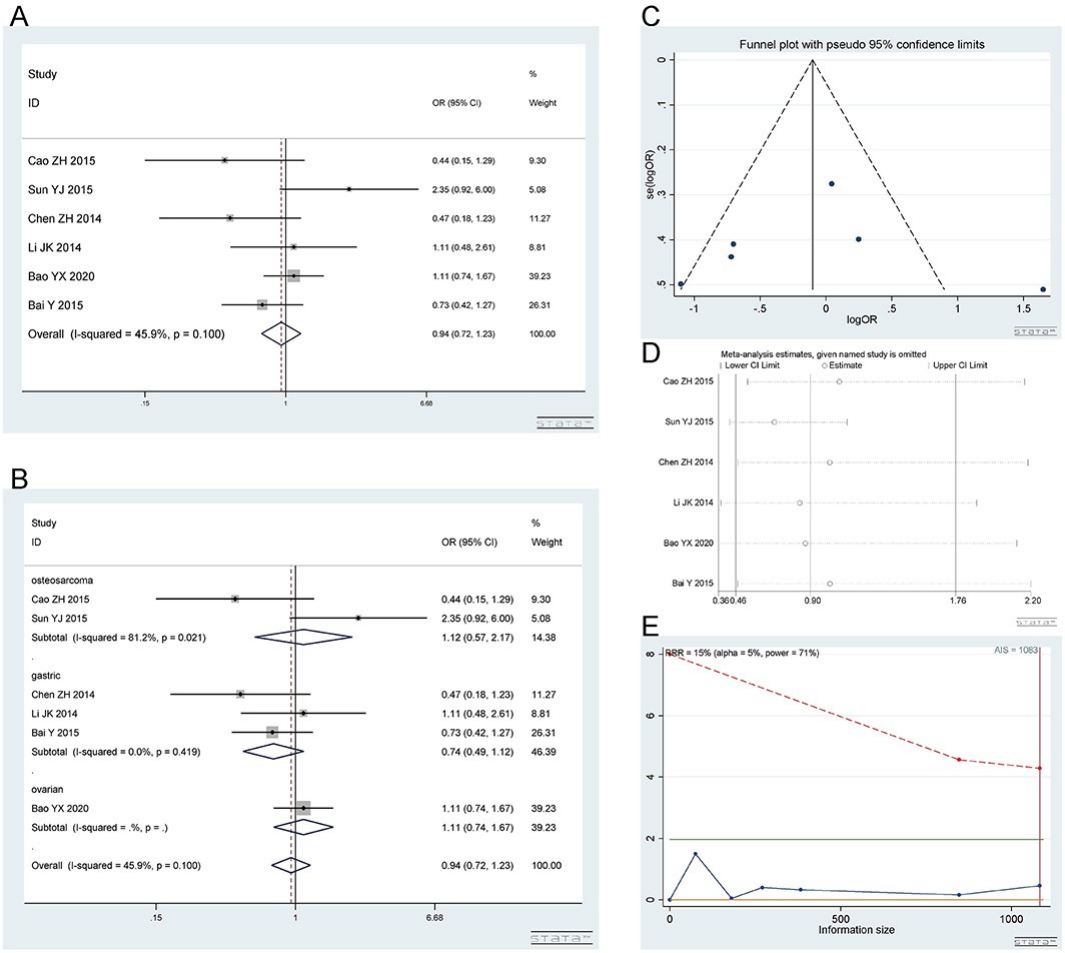
Meta-analysis of the response to platinum-based chemotherapy in the rs2298881 heterozygous model. (a) Result of the response to platinum-based chemotherapy in all cancer species. (b) Result of subgroup analysis of the response to platinum-based chemotherapy according to cancer species. (c) The funnel plot of publishing bias. (d) Result of the sensitivity analysis. (e) TSA on the response to platinum. The analysis included 1678 participants, which exceeded the required information size (n = 1083).

*2*.*2*.*2 Heterozygous model (CA vs CC)*. Number of studies and patients involved in the study as described in 2.2.1. The CA and CC genotypes showed no difference in platinum sensitivity. Heterozygous model (CA vs CC): I^2^ = 45.9%, fixed effect model was used, OR = 0.94, 95% CI(0.72,1.23), P = 0.653, as shown in Fig 6(A). In the subgroup analysis, the CA vs CC genotype was not related to platinum response in the gastric cancer [I^2^ = 42.2%, OR = 0.75, 95% CI (0.32,1.73), P = 0.495], osteosarcoma [I^2^ = 81.2%, OR = 1.12, 95% CI (0.57,2.17), P = 0.747] and ovarian cancer [OR = 1.11, 95% CI (0.74,1.67), P = 0.600] groups, as shown in Fig 6(B).

The funnel plot was basically symmetrical (Fig 6(C)). Begg’s test and Egger’s test showed that P = 0.462 and 0.860, respectively, indicating that there was no publication bias. The sensitivity analysis showed that the result was robust (Fig 6(D)). The TSA results showed that the enrolled studies were adequate (Fig 6(E)).

**Fig 7:**
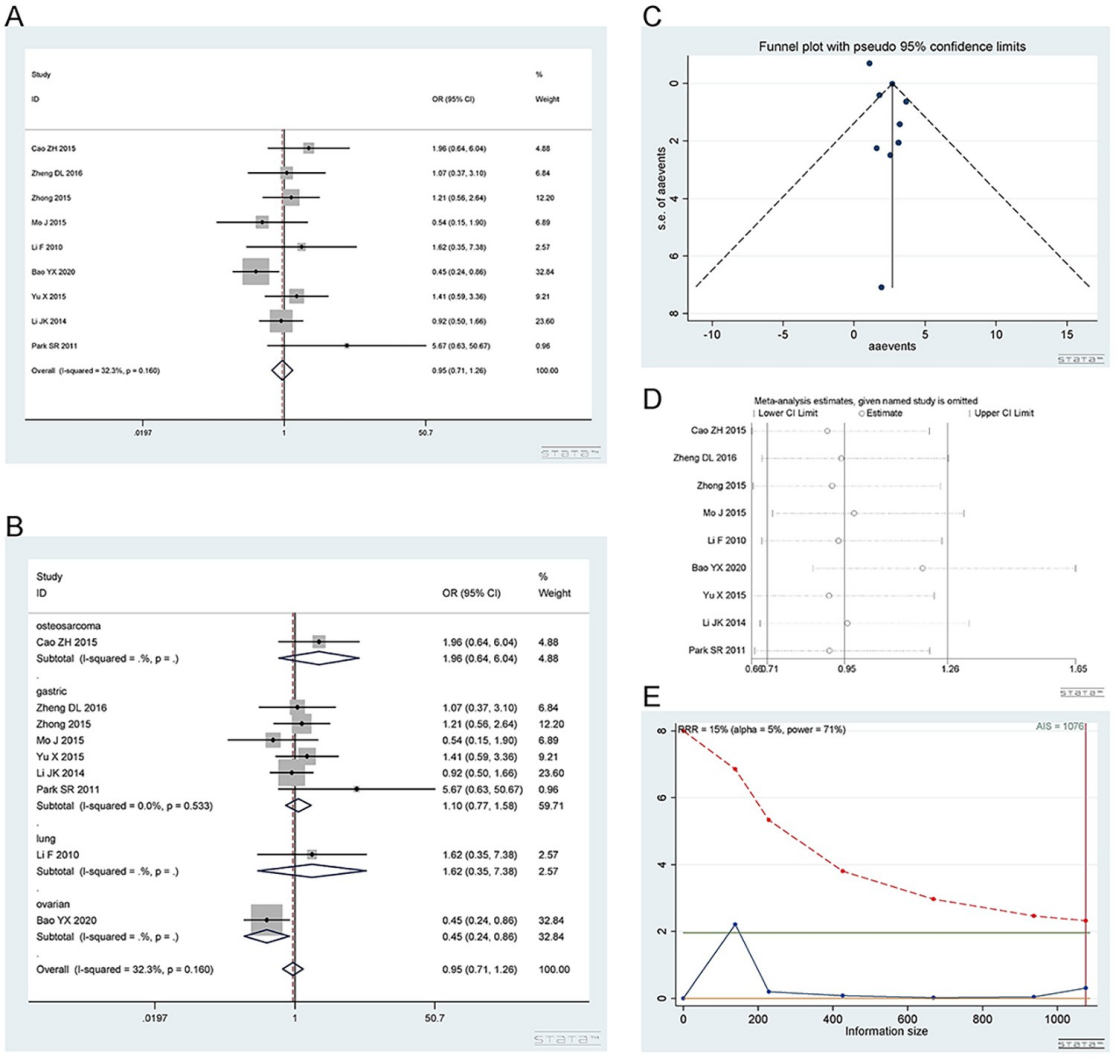
Meta-analysis of the response to platinum-based chemotherapy in the rs2298881 homozygous model. (a) Result of the response to platinum-based chemotherapy in all cancer species. (b) Result of subgroup analysis of the response to platinum-based chemotherapy according to cancer species. (c) The funnel plot of publishing bias. (d) Result of the sensitivity analysis. (e) TSA on the response to platinum. The analysis included 1678 participants, which exceeded the required information size (n = 1076).

*2*.*2*.*3 Homozygous model (AA vs CC)*. Number of studies and patients involved in the study as described in 2.2.1. The AA and CC genotypes showed no difference in platinum sensitivity. Homozygous model (AA vs CC): I^2^ = 76.2%, random effect model was used, OR = 0.90, 95% CI(0.46,1.76), P = 0.758, as shown in Fig 7(A). In the subgroup analysis, the AA vs CC genotype was not related to platinum response in the gastric cancer [I^2^ = 45.8%, OR = 0.69, 95% CI (0.36,1.30), P = 0.248], osteosarcoma [I^2^ = 93.3%, OR = 1.31, 95% CI (0.09,19.60), P = 0.843] and ovarian cancer [OR = 1.04, 95% CI (0.61,1.79), P = 0.873] groups, as shown in Fig 7(B).

The funnel plot was basically symmetrical (Fig 7(C)). Begg’s test and Egger’s test showed that P = 1.000 and 0.969, respectively, indicating that there was no publication bias. The sensitivity analysis showed that the result was robust (Fig 7(D)). The TSA results showed that the enrolled studies were adequate (Fig 7(E)).

#### 2.3 The rs3212986 A/C polymorphisms in ERCC1

**Fig 8:**
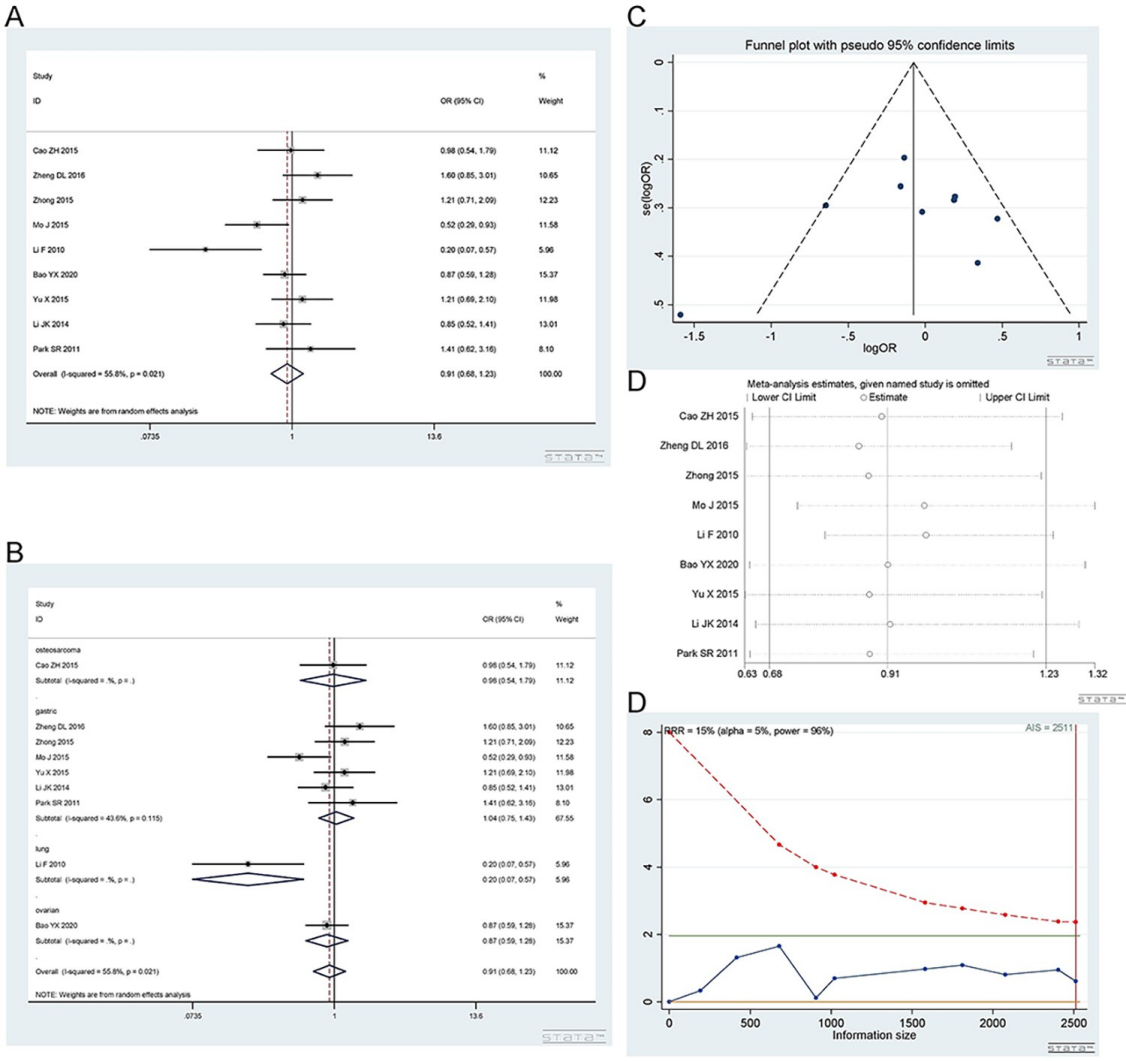
Meta-analysis of the response to platinum-based chemotherapy in the rs3212986 dominant model. (a) Result of the response to platinum-based chemotherapy in all cancer types. (b) Result of subgroup analysis of the response to platinum-based chemotherapy according to cancer type. (c) The funnel plot of publishing bias. (d) Result of the sensitivity analysis. (e) TSA on the response to platinum. The analysis included 2545 participants, which exceeded the required information size (n = 2511).

*2*.*3*.*1 Dominant model (CA+AA vs*. *CC)*. The rs3212986 polymorphism has a wild-type C allele and variant A allele. A total of 9 studies [16, 17, 20, 24, 25, 33–36], involving 2545 patients were included. There are 7 studies on gastric cancer, 1 on lung cancer, 1 on ovarian cancer and 1 on osteosarcoma. The CA+AA vs. CC phenotype showed no difference in response to platinum. Dominant model (CA+AA vs. CC): I^2^ = 65.8%, random effect model was used, OR = 0.91, 95% CI(0.68,1.22), P = 0.542, as shown in Fig 8(A). A subgroup analysis was subsequently carried out. In the subgroup analysis, the CA+AA vs. CC genotype was not related to platinum response in the gastric cancer [I^2^ = 56.9%, OR = 1.05, 95% CI (0.77,1.43), P = 0.772], osteosarcoma [OR = 1.10, 95% CI (0.62,1.96), P = 0.742] and ovarian cancer [OR = 0.71, 95% CI (0.50,1.02), P = 0.061] groups. The CA+AA vs. CC genotype was related to the response to platinum in lung cancer [OR = 0.23, 95% CI (0.10, 0.57), P = 0.002]. The results are shown in Fig 8(B). There was only one study in osteosarcoma, lung cancer and ovarian cancer.

The funnel plot was basically symmetrical (Fig 8(C)). Begg’s test and Egger’s test showed that P = 0.592 and 0.993, respectively, indicating that there was no publication bias. The sensitivity analysis showed that the results were robust (Fig 8(D)). The TSA results showed that the enrolled studies were adequate (Fig 8(E)).

**Fig 9:**
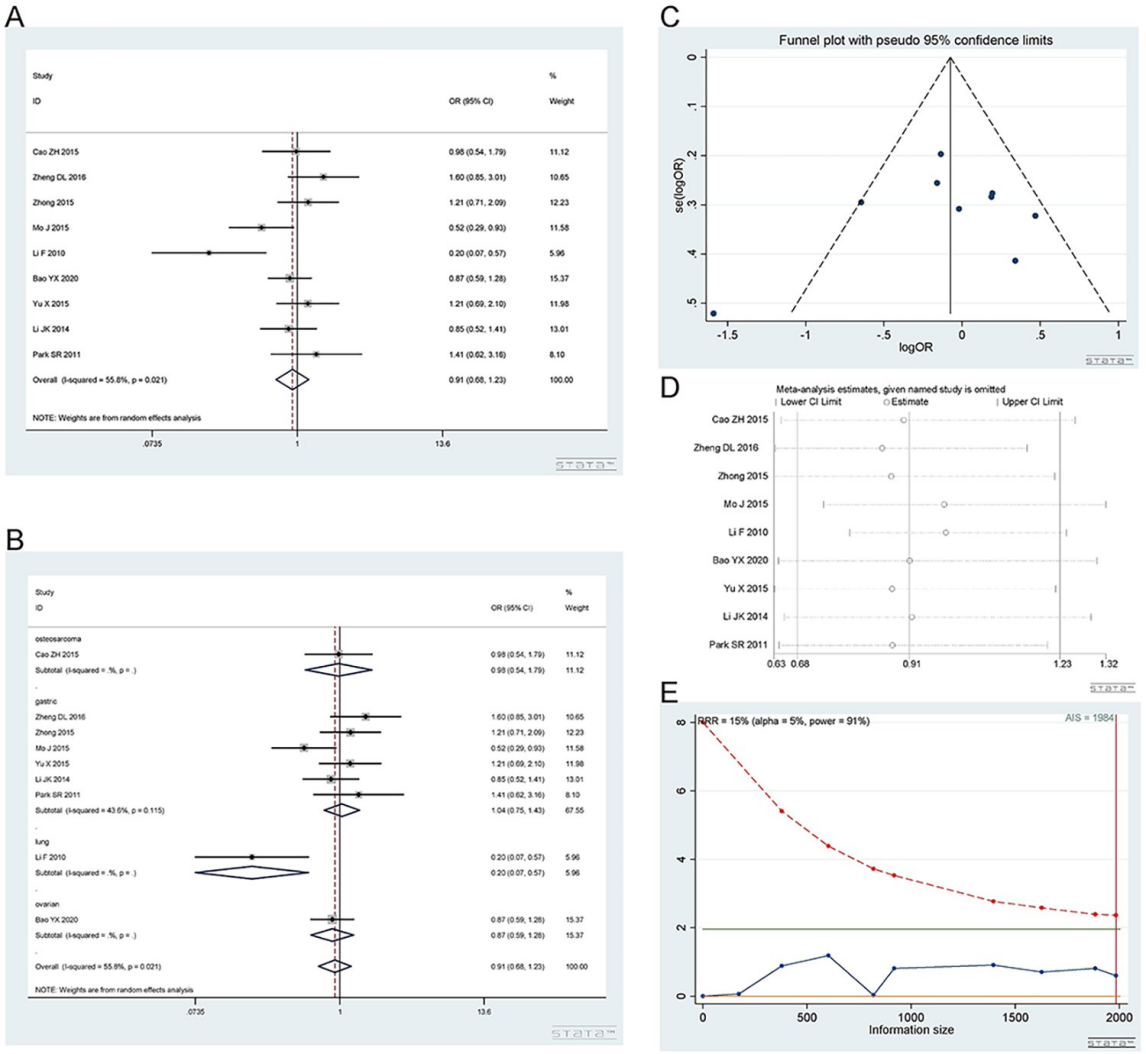
Meta-analysis of the response to platinum-based chemotherapy in the rs3212986 heterozygous model. (a) Result of the response to platinum-based chemotherapy in all cancer species. (b) Result of subgroup analysis of the response to platinum-based chemotherapy according to cancer species. (c) The funnel plot of publishing bias. (d) Results of the sensitivity analysis. (e) TSA on the response to platinum. The analysis included 2545 participants, which exceeded the required information size (n = 1984).

*2*.*3*.*2 Heterozygous model (CA vs CC)*. Number of studies and patients involved in the study as described in 2.3.1. The CA and CC genotypes showed no difference in response to platinum. Heterozygous model (CA vs CC): I^2^ = 55.8%, random effect model was used, OR = 0.91, 95% CI(0.68,1.23), P = 0.551, as shown in Fig 9(A). A subgroup analysis was subsequently carried out. In the subgroup analysis, the CA vs CC genotype was not related to platinum response in gastric cancer [I^2^ = 43.6%, OR = 1.04, 95% CI (0.75,1.43), P = 0.821], osteosarcoma [OR = 0.98, 95% CI (0.54,1.79), P = 0.949] or ovarian cancer [OR = 0.87, 95% CI (0.59,1.28), P = 0.489]. The CA vs CC genotype was related to the response to platinum in the lung cancer [OR = 0.20, 95% CI (0.07, 0.57), P = 0.002] groups, as shown in Fig 9(B).

The funnel plot was basically symmetrical (Fig 9(C)). Begg’s test and Egger’s test showed that P = 0.251 and 0.391, respectively, indicating that there was no publication bias. The sensitivity analysis showed that the result was robust (Fig 9(D)). The TSA results showed that the enrolled studies were adequate (Fig 9(E)).

**Fig 10:**
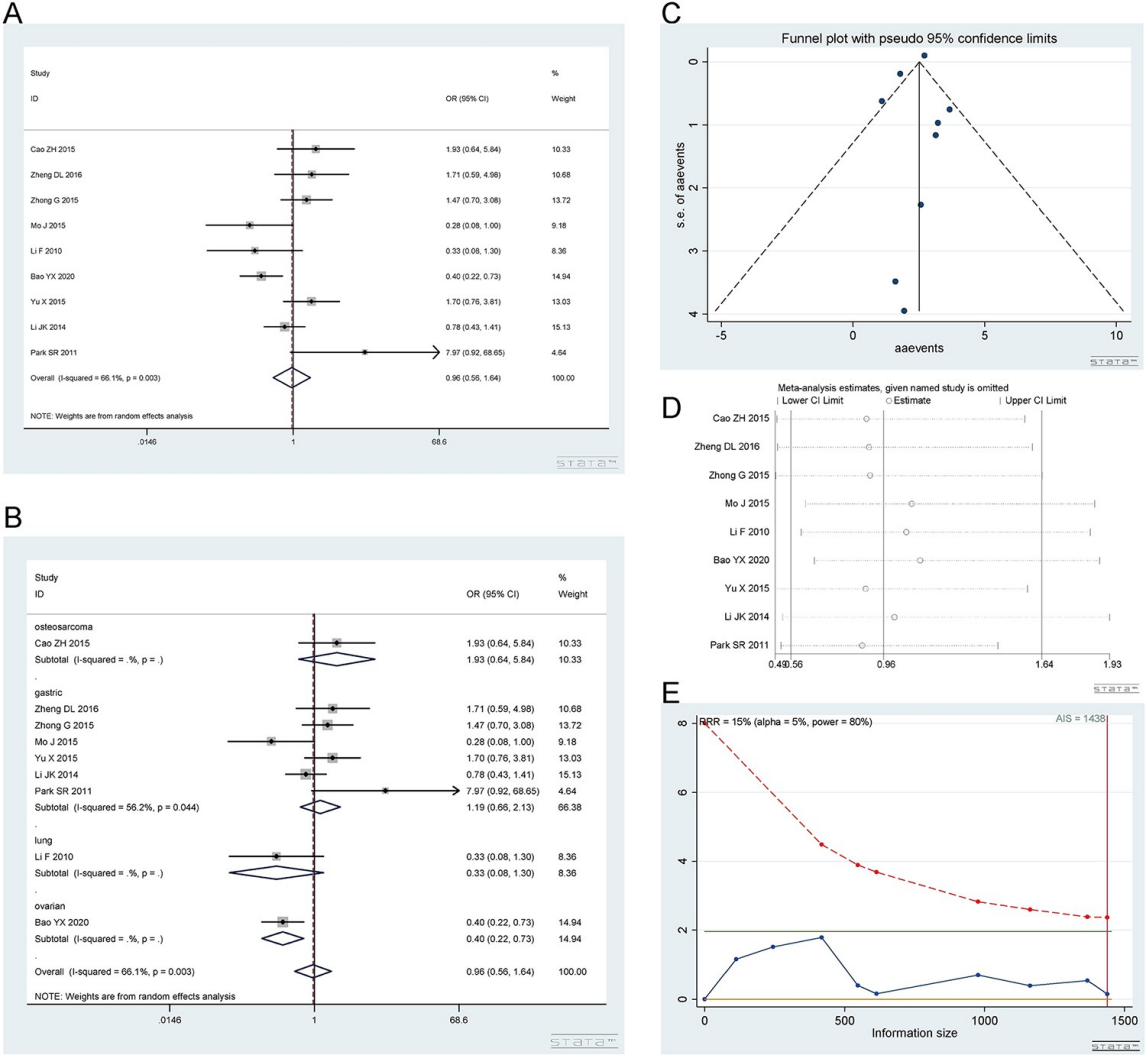
Meta-analysis of the response to platinum-based chemotherapy in the rs3212986 homozygous model. (a) Result of the response to platinum-based chemotherapy in all cancer species. (b) Result of subgroup analysis of the response to platinum-based chemotherapy according to cancer species. (c) The funnel plot of publishing bias. (d) Results of the sensitivity analysis. (e) TSA on the response to platinum. The analysis included 2545 participants, which exceeded the required information size (n = 1984).

*2*.*3*.*3 Homozygous model (AA vs CC)*. Number of studies and number of patients involved in the study as described in 2.3.1. The AA and CC genotypes showed no difference in response to platinum. Homozygous model (AA vs CC): I^2^ = 66.1%, random effect model was used, OR = 0.96, 95% CI(0.56,1.64), P = 0.883, as shown in Fig 10(A). In the subgroup analysis, the AA vs CC genotype was not related to platinum response in the gastric cancer [I^2^ = 56.2%, OR = 1.19, 95% CI (0.66,2.13), P = 0.562], osteosarcoma [OR = 1.93, 95% CI (0.64,5.84), P = 0.247] and lung cancer [OR = 0.33, 95% CI (0.08,1.30), P = 0.113] groups. The AA vs CC genotype was related to the response to platinum in the ovarian cancer [OR = 0.40, 95% CI (0.22, 0.73), P = 0.003] group. The above results are shown in Fig 10(B).

The funnel plot was basically symmetrical (Fig 10(C)). Begg’s test and Egger’s test showed that P = 0.466 and 0.336, respectively, indicating that there was no publication bias. The sensitivity analysis showed that the result was robust (Fig 10(D)). The TSA results showed that the enrolled studies were adequate (Fig 10(E)).

### 3. Overall survival in ERCC1 polymorphism

#### 3.1 The rs11615 polymorphism

**Fig 11:**
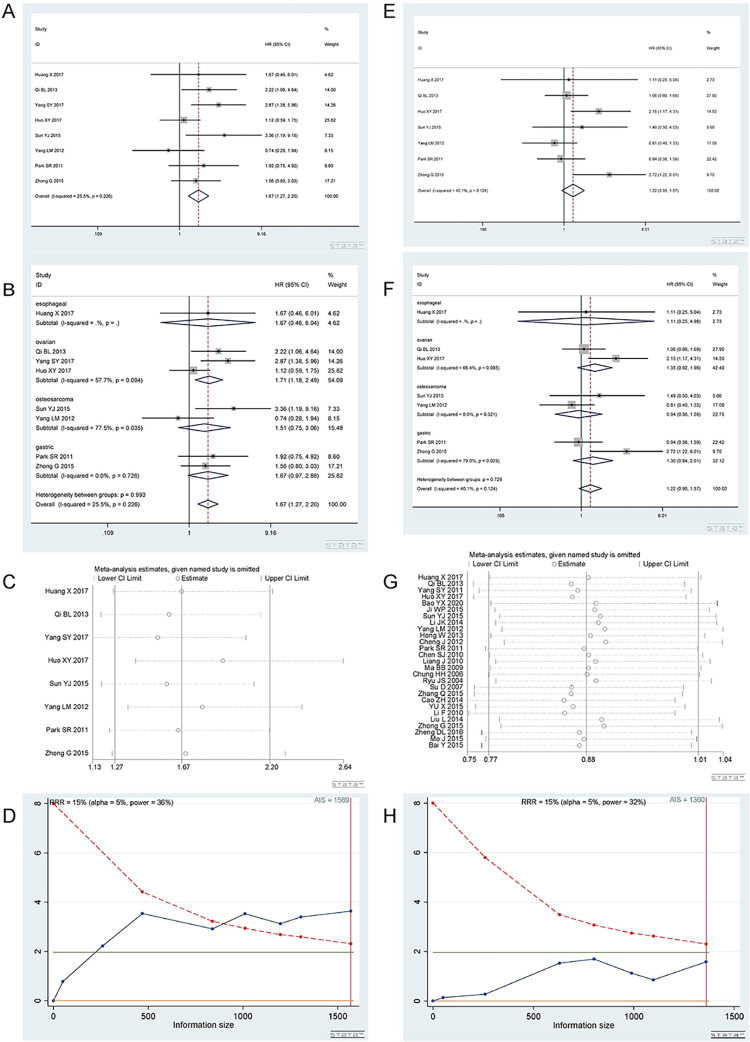
Meta-analysis of overall survival according to the rs11615 polymorphism. (a) Result of the overall survival of the homozygous model in all cancer species. (b) Result of subgroup analysis of the overall survival of the homozygous model according to cancer type. (c) Results of the sensitivity analysis. (d) TSA on the overall survival in homozygous model. (e) Result of the overall survival of the heterozygous model in all cancer type. (f) Result of subgroup analysis of the overall survival of the heterozygous model according to cancer type. (g) Result of the sensitivity analysis. (h) TSA of overall survival in the heterozygous model.

*3*.*1*.*1 Homozygous model (TT vs CC)*. A total of 8 studies [12–15, 19, 21, 24, 34] involving 1569 patients were included in the homozygous model. There were 3 studies on ovarian cancer, 2 on osteosarcoma, 2 on gastric cancer, and 1 on esophageal cancer. CC predicted longer overall survival than the TT genotype. Homozygous model (TT vs CC): I^2^ = 25.5%, fixed effect model was used, HR = 1.67, 95% CI(1.27,2.20), P<0.001, as shown in Fig 11(A). In subgroup analysis, TT vs CC genotype was not related to OS in esophageal cancer [HR = 1.67, 95%CI(0.46,6.04), P = 0.434], gastric cancer [I^2^ = 0%,HR = 1.67,95%CI(0.97,2.88), P = 0.064] and osteosarcoma [I^2^ = 77.5%,HR = 1.51,95%CI(0.75,3.06), P = 0.246] groups. The CC genotype was related to longer overall survival in ovarian cancer [I^2^ = 57.7%, HR = 1.71, 95% CI (1.18, 2.49), P<0.001], as shown in Fig 11(B). There was only one study in the esophagus.

Begg’s test and Egger’s test showed that P = 0.902 and 0.530, respectively, indicating that there was no publication bias. The sensitivity analysis showed that the result was reliable (Fig 11(C)). TSA showed that the AIS was 1569, and the sample size met the requirement (Fig 11(D)).

*3*.*1*.*2 Heterozygous model (CT vs CC)*. A total of 7 studies [12, 13, 15, 19, 21, 24, 34] involving 1360 patients were included in the homozygous model. Heterozygous model (CT vs CC): I^2^ = 40.1%, fixed effect model was used, HR = 1.22, 95% CI (0.95,1.57), P = 0.115, as shown in Fig 11(E). Subgroup analysis was subsequently carried out. In subgroup analysis, CT vs CC genotype was not related to OS in esophageal cancer [HR = 1.11, 95%CI(0.25,4.98), P = 0.892], osteosarcoma [I^2^ = 0%,HR = 0.94,95%CI(0.56,1.59), P = 0.824], gastric cancer [I^2^ = 79.0%, HR = 1.30, 95%CI(0.84,2.01), P = 0.247] and ovarian cancer [I^2^ = 66.4%,HR = 1.35,95%CI(0.92,1.98), P = 0.123] groups. The forest plot is shown in Fig 11(F). There was only one study in the esophageal cancer group.

Begg’s test and Egger’s test showed that P = 0.548 and 0.398, respectively, indicating that there was no publication bias. The sensitivity analysis showed that the results were not robust (Fig 11(G)). TSA showed that the AIS was 1360, and the sample size met the requirement (Fig 11(H).

#### 3.2 The rs2298881 A/C polymorphisms

**Fig 12:**
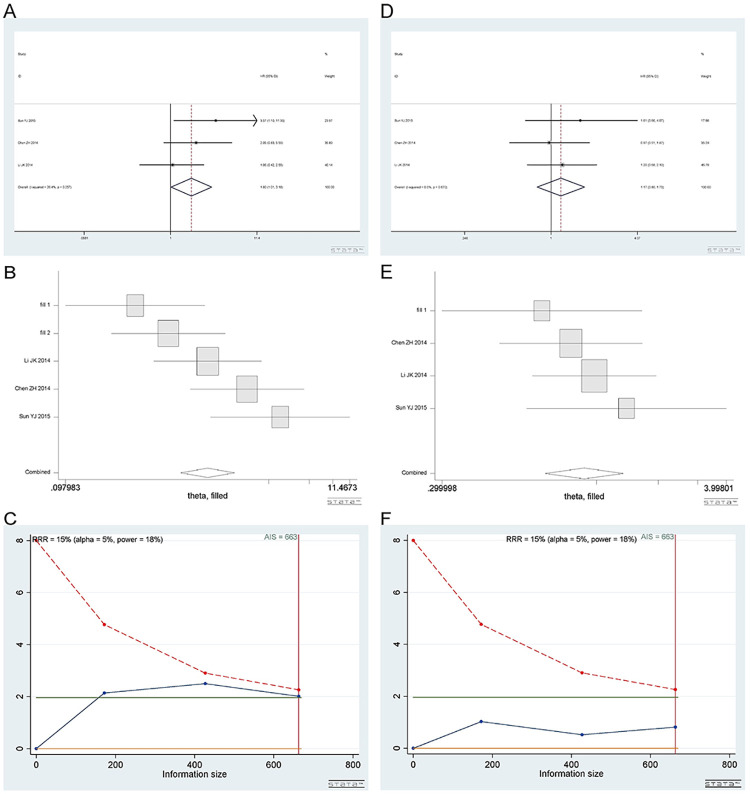
Meta-analysis of overall survival according to the rs2299981 polymorphism. (a) Result of the overall survival of the homozygous model. (b) Result of the overall survival of the homozygous model after meta-trim. (c) TSA on the overall survival in homozygous model. (d) Result of the overall survival of the heterozygous model. (e) Result of the overall survival of the heterozygous model after meta-trim. (f) TSA of overall survival in the heterozygous model.

*3*.*2*.*1 Homozygous model (AA vs CC)*. A total of 3 studies [19, 20, 38] were included in the homozygous model, involving 663 patients. There were 3 studies on gastric cancer, 2 on osteosarcoma and 1 on ovarian cancer. Homozygous model (AA vs CC): I^2^ = 26.4%, fixed effect model was used, HR = 1.80, 95% CI(1.01,3.18), P = 0.044, as shown in Fig 12(A). Because there were too few studies included, the meta-analysis was conducted again after the study was trimmed and completed to verify the sensitivity. HR = 1.06, 95% CI (0.68,1.67), P = 0.800, as shown in Fig 12(B). AA vs CC was not related to overall survival. TSA showed that the AIS was 663, and the sample size met the requirement (Fig 12(C).

*3*.*2*.*2 Heterozygous model (AC vs CC)*. Number of studies and number of patients involved in the study as described in 3.2.1. AC vs CC was not related to overall survival. Heterozygous model (AC vs CC): I^2^ = 0%, fixed effect model was used, HR = 1.17, 95% CI (0.80,1.73), P = 0.416, as shown in Fig 12(D). The result of the meta-analysis after trim and fill showed that HR = 1.10, 95% CI (0.77,1.56), P = 0.616 (Fig 12(E)). TSA showed that the AIS was 663, and the sample size met the requirement (Fig 12(F).

#### 3.3 The rs3212986 A/C polymorphisms

**Fig 13:**
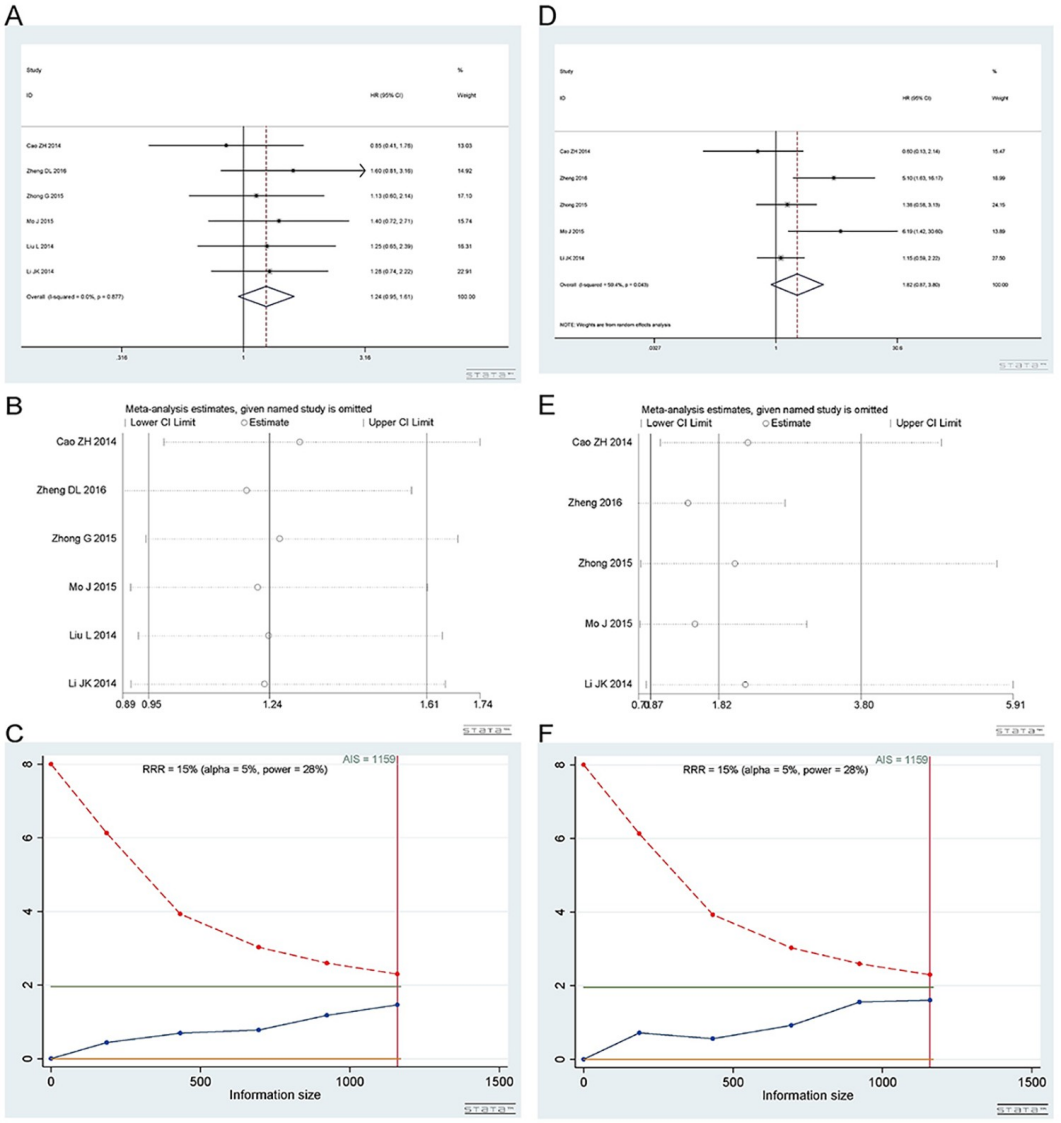
Meta-analysis of overall survival according to the rs3212986 polymorphism. (a) Result of the overall survival of the heterozygous model. (b) Result of sensitivity analysis in the heterozygous model. (c) TSA on the overall survival in heterozygous model. (d) Result of the overall survival of the homozygous model. (e) Result of sensitivity analysis in the homozygous model. (f) TSA of overall survival in the homozygous model.

*3*.*3*.*1 Heterozygous model (AC vs CC)*. A total of 5 studies [17, 20, 34–36] involving 1159 patients were included in the heterozygous model. There were 7 studies on gastric cancer, 1 on osteosarcoma, 1 on lung cancer, and 1 on ovarian cancer. AC vs CC was not related to overall survival. Heterozygous model (AC vs CC): I^2^ = 0%, fixed effect model was used, HR = 1.24, 95% CI (0.95,1.61), P = 0.108, as shown in Fig 13(A).

Begg’s test and Egger’s test showed P = 1.000 and 0.580, respectively, indicating that there was no publication bias. The sensitivity analysis showed that the result was reliable (Fig 13(B)). TSA showed that the AIS was 1159, and the sample size met the requirement (Fig 13(C)).

*3*.*3*.*2 Homozygous model (AA vs CC)*. Number of studies and number of patients involved in the study as described in 3.3.1. Homozygous model (AA vs CC): I^2^ = 59.4%, random effect model was used, HR = 1.82, 95% CI(0.87,3.80), P = 0.109, as shown in Fig 13(D).

Begg’s test and Egger’s test showed that P = 0.806 and 0.422, respectively, indicating that there was no publication bias. The sensitivity analysis showed that the result was reliable (Fig 13(E)). TSA showed that the AIS was 1159, and the sample size met the requirement (Fig 13(F)).

## Discussion

The study explored the effects of ERCC1 rs11615, rs2298881 and rs3212986 polymorphisms on platinum responsiveness and OS. Platinum responsiveness may reflect short-term effects, whereas the OS reaction can reflect long-term effects. These two results are correlated, but not necessarily consistent, and therefore both need to be evaluated. In ERCC1 rs11615, the CT and CC genotypes predicted a better response to platinum in ovarian cancers than the TT genotypes. Polymorphisms were also associated with OS. The CC genotype was associated with longer overall survival in ovarian cancer patients than in TT patients. In ERCC1 rs2298881 and rs3212986, the A/C polymorphism was not related to the response to platinum and OS.

The ERCC1 protein is an excision nuclease that acts within the NER pathway and is involved in platinum metabolism. There are many mechanisms of platinum resistance, and NER is the main mechanism leading to moderate resistance [39]. From the above results, it is easy to see that the effect of ERCC1 polymorphism on platinum responsiveness is related to cancer type. This may be related to the influence of the NER pathway on the occurrence and development of different cancers. NER is a key pathway for dysregulated DNA repair in esophageal adenocarcinoma [40], ovarian cancer [41] and gastric cancer [8]. ERCC1 rs11615, rs229881 and rs3212986 are the most intensively studied SNPs. ERCC1 is a key rate-limiting enzyme in the multistep NER process. ERCC1, in collaboration with the XPF protein, is involved in DNA lesion recognition. Therefore, functional ERCC1 SNPs may contribute directly to phenotypes of drug sensitivity by modifying the function of related genes and reflecting platinum sensitivity as an innate trait [8].

The TT subtype of rs11615 was associated with platinum resistance, whereas CT and TT containing the T allele were associated with shorter OS. This result can be explained by biological significance. The rs11615 T allele of the ERCC1 polymorphism was found to be associated with high mRNA expression of the corresponding gene [42]. Functional studies confirmed the substantial effect of the ERCC1 rs11615C>T SNPs on the phenotype of NER capacity [42–44], which leads to enhanced DNA repair capacity and even to the development of platinum resistance.

The reasons for this significant ethnic difference are unclear which may be due to significant race-specific differences in linkage disequilibrium (LD) between rs11615 SNP and other nearby alleles that may cause platinum resistance [45]. The difference results in a possible lower prognostic and predictive value of the rs11615 SNP in African Americans and Africans than in Caucasians and Asians. In the Asian (CHB+JPT) population, at least five upstream polymorphisms within or directly adjacent to the ERCC1 gene were associated with rs11615. In addition to altering ERCC1 expression, phenotypic alterations in rs11615 may also lead to alterations in adjacent genes [46].

Our study not only analyzed the platinum sensitivity and prognosis of different SNPs but also compared different cancers horizontally. It can be seen that different cancers and different SNPs have varying effects on platinum sensitivity and prognosis. Ovarian cancer identified in this study was closely related to ERCC1 gene polymorphisms. Ovarian cancer patients do benefit from DNA damage repair pathway inhibitors, such as Rucaparib [47–49], Olaparib [50–52] and Niraparib [53–56]. These drugs target DNA damage repair (DDR) pathways, such as BRCA1/2 mutation and homologous recombination deficiency. In addition, HRR deficiency can also cause hereditary ovarian cancer [57]. All the above evidence suggests that ovarian cancer is a genomically unstable tumor, which may be the reason why the NER pathway represented by ERCC1 affects it more significantly. The lack of a specific DDR pathway may lead to mutation or chromosomal rearrangement and other consequences, promoting genomic instability and tumorigenesis [58]. Other similar studies have examined the relationship between only one SNP and one type of cancer. Our study focused on different cancer types and races. The limitation of this study is that some results may have boundary effects. But all of the results are reliable through sensitivity analysis. We did not search for documents published in other languages except English and Chinese, which may lead to bias due to language restrictions. In clinical practice, clinicians may prefer to select chemotherapy schemes other than platinum containing chemotherapy for patients with ERCC1 polymorphism rs11615 TT genotype, such as early use of PARP inhibitors and anti-angiogenesis, especially in ovarian cancer.

## Conclusion

The ERCC1 rs11615 CT and CC genotypes predicted a better response to platinum than the TT genotype in esophageal and ovarian. The CC genotype was related to longer overall survival than TT in ovarian cancer. The ERCC1 rs2298881 and rs3212986 A/C polymorphisms were not related to the response to platinum and OS.

## Supporting information

S1 Checklist PRISMA 2020 checklist. (DOCX)

S1 Table Literature search strategy. (DOCX)
