# Supplementary material for: Effect of ERCC1 polymorphisms on the response to platinum-based chemotherapy: A systematic review and meta-analysis based on Asian population
Source: PLoS One. 2023 May 4;18(5):e0284825. doi: 10.1371/journal.pone.0284825 (PMC10159199; doi:10.1371/journal.pone.0284825)
Supplement: S1 Table — (DOCX) [file pone.0284825.s002.docx]

| No. | database | Literature search strategy |
| --- | --- | --- |
| 1 | Pubmed | (((ERCC1[Title/Abstract]) OR ("Ercc1 protein, Arabidopsis" [Supplementary Concept])) AND (((("Platinum"[Mesh]) OR "Cisplatin"[Mesh]) OR "lobaplatin" [Supplementary Concept]) OR ((((((((platinum[Title/Abstract]) OR (carboplatin[Title/Abstract])) OR (cisplatin[Title/Abstract])) OR (oxaliplatin[Title/Abstract])) OR (lobaplatin[Title/Abstract])) OR (nedaplatin[Title/Abstract])) OR (resistant[Title/Abstract])) OR (chemotherapy response[Title/Abstract])))) AND (("Polymorphism, Genetic"[Mesh]) OR (((polymorphism[Title/Abstract]) OR (SNP[Title/Abstract])) OR (genetic variants[Title/Abstract]))) |
| 2 | CNKI | (SU=ERCC1 OR ERCC-1) AND ((SU=polymorphism OR SU=SNP OR SU=genetic variant) AND (SU=platinum OR SU=carboplatin OR SU=cisplatin OR SU=oxaliplatin OR SU=lobaplatin OR SU=nedaplatin) |
| 3 | VIP | M=ERCC1 AND (M=polymorphism OR M=genetic variant OR M=SNP ) AND (M=Platinum OR M=carboplatin OR M=cisplatin OR M=oxaliplatin OR M=lobaplatin OR M=nedaplatin) |
| 4 | CBM | ERCC1 AND (polymorphism OR genetic variant OR SNP ) AND (Platinum OR carboplatin OR cisplatin OR oxaliplatin OR lobaplatin OR nedaplatin) |
| 5 | Wanfang | Subject:(ERCC1) and (subject:( polymorphism) or subject:(genetic variant) or subject:(SNP) and (subject:(Platinum) or subject:(carboplatin) OR subject:(cisplatin) or subject:(oxaliplatin) or subject:(lobaplatin) OR subject:(nedaplatin)) |
| 6 | Embase | ercc1:ti,ab,kw AND (polymorphism:ti,ab,kw OR 'genetic variant':ti,ab,kw OR snp:ti,ab,kw) AND (Platinum:ti,ab,kw OR 'carboplatin':ti,ab,kw OR 'cisplatin':ti,ab,kw 'oxaliplatin':ti,ab,kw OR 'lobaplatin':ti,ab,kw OR 'nedaplatin':ti,ab,kw) |
| 7 | Scopus | ( TITLE-ABS-KEY ( ercc1 AND polymorphism ) AND TITLE-ABS-KEY ( platinum OR carboplatinum OR cisplatin OR oxaliplatin OR lobaplatin OR nedaplatin ) AND TITLE-ABS-KEY ( cohort OR case AND control ) ) |
| 8 | Cochrane Library | #1：(ERCC1):ti,ab,kw  #2：(polymorphism OR SNP OR genetic variant):ti,ab,kw  #3：(platinum OR carboplatinum OR cisplatin OR oxaliplatin OR lobaplatin OR nedaplatin) :ti,ab,kw  #4：#1 AND #2 AND #3 |

S1 Table Literature search strategy
